# B-type natriuretic peptide release and left ventricular filling pressure assessed by echocardiographic study after subarachnoid hemorrhage: a prospective study in non-cardiac patients

**DOI:** 10.1186/cc7891

**Published:** 2009-05-20

**Authors:** Eric Meaudre, Christophe Jego, Nadia Kenane, Ambroise Montcriol, Henry Boret, Philippe Goutorbe, Gilbert Habib, Bruno Palmier

**Affiliations:** 1 Department of Anesthesiology and Critical Care, Hôpital d'Instruction des Armées Sainte-Anne, Boulevard Sainte-Anne, Toulon, BP 20545 – 83041, Cedex 9, France; 2 Department of Cardiology, Hôpital d'Instruction des Armées Sainte-Anne, Boulevard Sainte-Anne, Toulon, BP 20545 – 83041, Cedex 9, France; 3 Department of Cardiology, Centre Hospitalo-Universitaire de la Timone, 264 Rue Saint-Pierre, Marseille, 13385, Cedex 5, France

## Abstract

**Introduction:**

Serum B-type natriuretic peptide (BNP) is frequently elevated after subarachnoid hemorrhage (SAH), but whether this high BNP level is related to transient elevation of left ventricular filling pressure (LVFP) is unknown. However, in patients with preexistent cardiac pathologies, it is impossible to differentiate between BNP elevation caused by chronic cardiac abnormalities and BNP related to acute neurocardiac injury.

**Methods:**

All adult patients with SAH admitted to our intensive care unit were eligible. Patients were excluded for the following reasons: admission >48 hours after aneurysm rupture, pre-existing hypertension, or cardiac disease. Levels of BNP and cardiac troponin Ic were measured daily for 7 days. Echocardiography was performed by a blinded cardiologist on days 1, 2, and 7. Doppler signals from the mitral inflow, tissue Doppler, and the color M-mode–derived flow propagation velocity (FPV) were obtained to assess echo-estimated LVFP.

**Results:**

During a 3-year period, sixty-six consecutive patients with SAH were admitted. Thirty one patients were studied. The BNP level was >100 ng/L in 25 patients (80%) during the first 3 days, with a peak on day 2 (median, 126 ng/L) followed by a gradual decrease (median variation days 1 to 7, 70%). All patients had an ejection fraction >50%. Early transmitral velocity/tissue Doppler mitral annular early diastolic velocity was low: 5.4 (± 1.5) on day 1, 5.8 (± 1.2) on day 2, and 5.1 (± 0.9) on day 7. Early transmitral velocity/FPV was also low: 1.27 (± 0.4), 1.25 (± 0.3), and 1.1 (± 0.2) on days 1, 2, and 7, respectively. Cardiac troponin Ic levels ranged from 0 to 3.67 μg/L and were correlated with BNP (*r *= 0.63, *P *< 0.01).

**Conclusions:**

BNP rises gradually over two days and return to normal within a week after SAH. Its release is associated with myocardial necrosis, but is unrelated to elevated LVFP assessed by echocardiography.

## Introduction

Serum plasma B-type natriuretic peptide (BNP) is a global indicator of left cardiac dysfunction. Recent reports have shown the contribution of left ventricular (LV) diastolic function to plasma BNP levels and the usefulness of BNP in the diagnosis of diastolic dysfunction [1]. Stretch of cardiomyocytes due to elevated filling pressures is reported to be the most important stimulus of BNP regulation [2]. Doppler echocardiography, color flow imaging, and myocardial tissue imaging can assess intrinsic diastolic function and estimate left ventricular filling pressure (LVFP) or pulmonary capillary wedge pressure with accuracy over a wide range of ejection fraction (EF) [3,4], including normal EF [5].

In patients with subarachnoid hemorrhage (SAH), the BNP level increases soon after aneurysm rupture and returns to baseline in one to two weeks [6-8]. The source of BNP release remains controversial [9]. However, the most likely cause of BNP increase after SAH is cardiac injury [10]. Cardiac injury is a well-recognized phenomenon after SAH and results in ECG changes [11], serum elevation of troponin Ic (cTi) [12,13], and LV systolic and diastolic dysfunction [14]. A cardiac source of BNP is also supported by a recent study demonstrating that cardiac injury and dysfunction occurring early after SAH are associated with elevated plasma BNP levels [10].

To answer a fundamental question, the hypothesis of the present study was that BNP elevation after SAH is triggered by a transient elevation of LVFP due to diastolic dysfunction. Nevertheless, in patients with heart disease it is impossible to know the baseline levels of BNP. Consequently, it is impossible to differentiate between BNP elevation caused by pre-existent chronic diastolic dysfunction with elevated filling pressures, and BNP increase in parallel with acute cardiac dysfunction caused by SAH. Therefore, this study was strictly limited to patients without pre-existing cardiac disease and without history of chronic hypertension, which is frequent before aneurysm rupture [15] and may be responsible for pre-existing diastolic dysfunction and BNP elevation.

The aim of this prospective cohort study of recent SAH patients (<48 hours) was to accurately quantify the incidence, time course, and recovery patterns of BNP and LVFP by using serial echocardiographic measurements during the first week after aneurysm rupture.

## Materials and methods

### Study design

This study was conducted in the intensive care unit (ICU) of the Military Teaching Hospital Sainte-Anne during a 36-month period between June 2004 and June 2007. The Military Teaching Hospital Sainte-Anne is the only neurosurgical hospital in the region of Var, which has a population of one million inhabitants. The study was approved by our local ethics committee, and all patients or next of kin provided written, informed consent.

Eligibility criteria for inclusion were the following: SAH related to a ruptured aneurysm documented by angiography; age over 18 years; and sinus rhythm 60 to 100 beats/min. Patients with chronic hypertension (history, antihypertensive treatment), heart disease such as cardiomyopathy, or prior myocardial infarction or atrial fibrillation (history, electrocardiogram, treatment) were not included. Patients, families, or referring physicians were interviewed to determine the date and nature of the first signs or symptoms which were clearly those of SAH. If delay from first sign or symptom to arrival at our ICU was more than 48 hours after aneurysm rupture symptoms, patients were not included. Patients who died before day 7 were excluded because of lack of parameters concerning the evolution of diastolic parameters and echo-estimated LVFP.

### Patients, management, and clinical data collection

All patients were admitted to our unit for a seven-day period and were managed according to the recommendations of the French Society for Anesthesia and Intensive Care [16]. In particular, the culprit cerebral aneurysm identified by angiography was treated as soon as possible by either endovascular coiling or neurosurgical clipping, depending on individual anatomy. All patients underwent transcranial Doppler evaluation once a day.

Conscious patients were managed with bed rest, continuous infusion of nimodipine at a rate of 2 mg/hour, phenytoine, analgesia (paracetamol, nefopam), and a proton pump inhibitor. Management of comatose patients included sedation, ventilation, enteral nutrition, nimodipine, and monitoring of intracranial pressure in the case of intracranial hypertension.

All patients were managed according to a standard protocol. Prophylactic hypervolemia was not used, but, on the contrary, our protocol was rather restrictive to avoid BNP elevation as a result of iatrogenic volume overload from therapeutic hypervolemia. During the first seven days, each patient received isotonic saline intravenous fluid ranging from 30 to 40 ml/kg/day. Fluid balance was calculated daily. Measurements of natremia and sodium balance were performed daily. If necessary, treatment of elevated intracranial pressure included mannitol, but not hypertonic saline. In patients with vasospasm, hematocrit target (30 to 35%) was employed for hypervolemia using additional intravenous infusion of isotonic saline for the purpose of intravascular volume expansion. The mean arterial blood pressure was maintained at a mean arterial pressure of 100 to 110 mmHg.

When intracranial hypertension occurred, norepinephrine was used to maintain cerebral perfusion pressure above 65 mmHg. Vasospasm was managed by moderate hypervolemic hypertension and intracranial angioplasty when possible.

Clinical and demographic data including age, sex, body mass index, and body surface area were collected. The fluid balance (fluid intake – urine volume – insensible losses) was calculated at 24-hour intervals. Insensible losses per day were estimated at 700 ml for all patients. Creatinine clearance was computed from creatinine excretion in a 24-hour urine collection and a single measurement of serum creatinine on day 2.

Aneurysm location was noted, and the neurological status was assessed at the time of admission and graded according to the World Federation of Neurosurgical Societies (WFNS) scale and the Fisher score. The presence or absence of cerebral vasospasm by imaging (transcranial Doppler and cerebral angiography) during the seven days was noted. In addition, the use of norepinephrine to maintain arterial pressure or cerebral perfusion pressure during the first three days was recorded. Data regarding aneurysmal treatment and neurological events (rebleeding, hydrocephalus, vasospasm) were recorded. Hyponatremia was defined as a sodium level of less than 135 mEq/L for at least two consecutive days.

### ECG and cardiac troponin Ic

A 12-lead ECG was performed daily for seven days. The ECGs were considered abnormal if the T wave was inverted or flattened, the S-T segment was elevated or depressed, the QT interval was prolonged, or arrhythmia was present.

Measurement of cTi was performed daily for seven days in all patients. The serum cTi levels were measured by ELISA (reference range for upper limit of normal, 0.14 μg/l; lower limit of detection, 0.04 μg/l) with a Siemens^® ^(Deerfield, IL, USA) analyzer.

### BNP determination

Arterial blood was drawn daily for seven days from patients and placed in a Vacutainer tube containing potassium EDTA. Within 30 minutes, the blood was placed on a Triage B-Type Natriuretic Peptide test slide (Biosite^® ^Diagnostics, San Diego, CA, USA) and analyzed in the Biosite MeterPlus machine, a point-of-care test based on fluorescence immunoassay. The test has a range of 5 to 5000 ng/l.

### Echocardiography and Doppler

Transthoracic echocardiography was performed on days 1 (day of admission), 2, and 7 with an ACUSON CV 70^® ^ultrasound system (Siemens^® ^CO, Erlangen, Germany) equipped with 2.5-MHz transducers. All Doppler echocardiography studies were performed by a single experienced cardiologist blinded to all clinical, hemodynamic, and BNP data.

Patients were imaged in the supine position. Two-dimensional images were obtained in the standard parasternal and apical views. All echocardiographic data were averaged from three to five end-expiratory cycles. Left ventricular and left atrial dimensions were measured according to the recommendations of the American Society for Echocardiography. Left ventricular mass was calculated by Devereux's formula and indexed for body-surface area. LVEF was measured by Simpson's method. An LVEF more than 50% was defined as normal; an LVEF less than 50% was defined as reduced.

**Figure 1:**
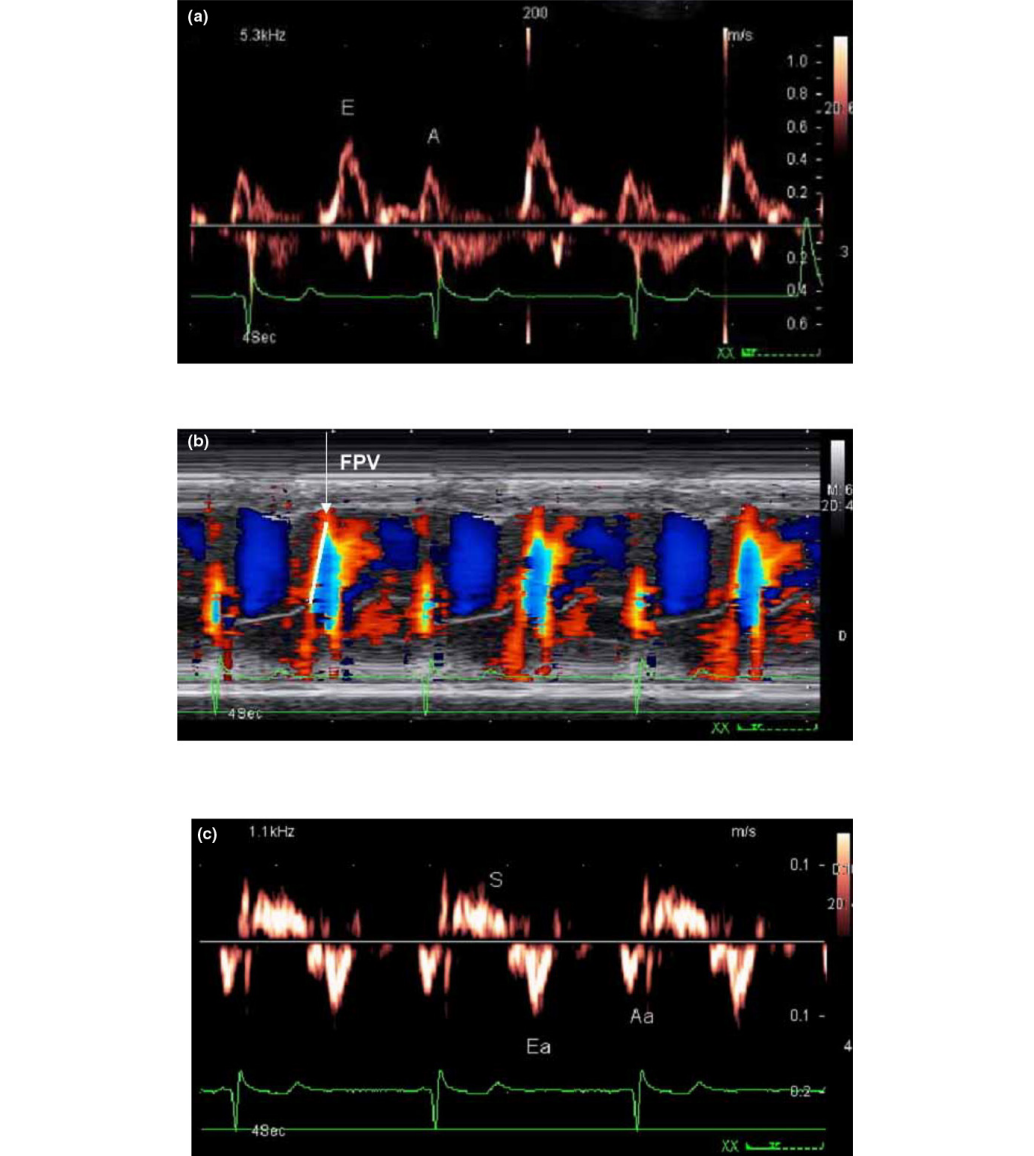
Echocardiographic parameters to estimate LV filling pressures (a) Mitral inflow, (b) color M-mode-derived flow propagation velocity (FPV), and (c) Tissue Doppler velocities at the lateral corners of the mitral annulus

All Doppler recordings were obtained at a sweep speed of 100 mm/s. Pulsed Doppler was used to record transmitral flow in the apical four-chamber view. Tissue Doppler velocities were acquired at a lateral annular site. Mitral inflow measurements included early peak (E) and late peak (A) velocities, E/A ratio, and deceleration time (DT) of E velocity. These measurements were analyzed as described previously [4]. Color M-mode-derived flow propagation velocity (FPV) was measured as the slope of the linear component of the color border produced by propagation of E velocity into the left ventricle past the mitral valve tips [17]. On tissue Doppler imaging recordings, early diastolic velocities (Ea) were measured. The combined indices E/FPV [17] and E/Ea [4] were computed (Figure 1). Isovolumic relaxation time (IVRT) was measured from the end of aortic flow to the onset of mitral inflow after placing the 5 mm pulsed Doppler sample volume between the mitral valve and the LV outflow in an apical five-chamber view. The systolic pulmonary artery pressure (PAP) was estimated using continuous-wave Doppler ultrasound measurement of the peak velocity of a tricuspid regurgitant jet.

### Statistical analysis

Statistical analysis was performed using SPSS version 15.0 (SPSS Inc., Chicago, IL, USA). Continuous variables were expressed as mean ± standard deviation or as median with interquartile range. The non-parametric Mann-Whitney U test was used to compare two groups. Correlations between parameters were calculated by using Spearman's correlation coefficient. For all tests, a *P *< 0.05 was considered significant.

## Results

**Figure 2:**
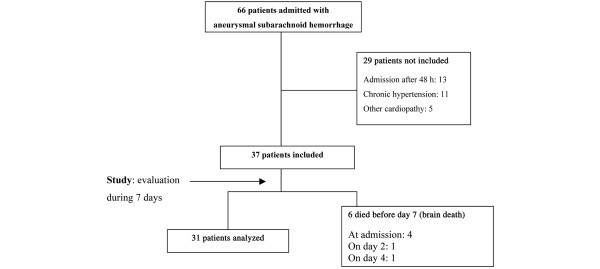
Flow diagram of subarachnoid hemorrhage patients from admission to day 7

During the study period, 66 consecutive patients were admitted to our ICU with SAH related to a ruptured aneurysm that was documented by angiography. Among them, 29 patients were excluded from the study. Six patients died during the first week and were excluded from the final analysis. Therefore, data from 31 patients were analyzed (Figure 2).

### Patients characteristics, fluid, and sodium balance

Patient characteristics and clinical events are shown in Tables 1 and 2. All six cases of vasospasm occured after day 5. Fuid balance, sodium balance, and natremia until day 4 are shown in Table 3. Hyponatremia was present in three cases. The median daily fluid balance was negative until day 7. The sodium balance was close to zero until day 7. The sodium and fluid balance were not different in patients with BNP more than 100, 150, or 250 ng/L. Renal function was normal: mean serum creatinine of 56 (± 15) μmol/L and mean measured creatinine clearance of 140 (± 60) ml/min.

**Table 1:** Clinical characteristics

Age, years (mean ± SD)	48 (± 12)
Female sex, n (%)	20 (65%)
Body mass index (mean ± SD)	22.8 (± 3.2)
Body surface area, m^2 ^(mean ± SD)	1.70 (± 0.17)
Fisher scale (1/2/3/4), n (%)	1 (3%)/10 (32%)/6 (20%)/14 (45%)
WFNS score (1/2/3/4/5), n (%)	14 (45%)/9 (30%)/2 (6%)/2 (6%)/4 (13%)
Aneurysm position, n (%)	
ICA	6 (19%)
MCA	9 (30%)
AComA/ACA	10 (32%)
BA	2 (6%)
PCA	1 (3%)
VA	3 (10%)

ACA = anterior cerebral artery; AcomA = anterior communicating artery; BA = basilar artery; ICA = internal carotid artery; MCA = middle cerebral artery; PCA = posterior cerebral artery; SD = standard deviation; VA = vertebral artery; WFNS = World Federation of Neurosurgical Societies.

**Table 2:** **Clinical events until day **7

ECG abnormalities, n (%)	11 (36%)
Coiled, n (%)	29 (94%)
on day 1	23
on day 2	5
on day 6	1
Craniotomy, n (%)	2 (6%)
on day 1	2
Vasospasm, n (%)	6 (19%)
Rebleeding, n (%)	1 (3%)
Hydrocephalus (derivated), n (%)	13 (42%)
Norepinephrine (used during days 1–3)	13 (42%)
Mechanical ventilation (day 1/day 7), n (%)	15 (48%)/5 (16%)

**Table 3:** Natremia (mean ± standard deviation), fluid balance, and sodium balance (median, interquartile range) during the first four days.

	Day 1	Day 2	Day 3	Day 4
Natremia (mmol/L)	140 ± 5	141 ± 7	142 ± 7	142 ± 8

Fluid balance (mL)	-750 (-975 to 275)	-650 (-1475 to 650)	-500 (-1250 to 100)	-700 (-1100 to 400)
Sodium balance (mEq)	136 (-59 to 221)	1 (-102 to 182)	-34 (-136 to 136)	-51 (-144 to 41)

### BNP time course

**Figure 3:**
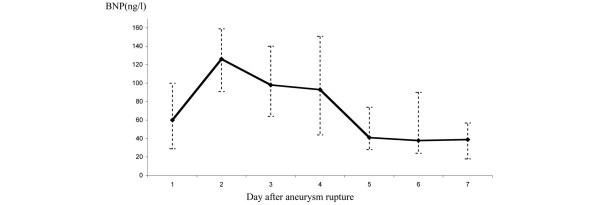
Daily median B-type natriuretic peptide (BNP) levels in 31 subarachnoid hemorrhage (SAH) patients without pre-existing chronic hypertension or cardiac disease.  Error bars indicate confidence intervals.

Twenty-five patients (80%) had a BNP level of more than 100 ng/L during the first three days. The peak BNP level was observed on day 2, with a median level of 126 ng/L (interquartile range, 53 to 202 ng/L; Figure 3). In four patients, no BNP increase was observed. The median variation in BNP (between a peak on day 1 or 2 and day 7) was 70% (interquartile range, 41 to 92%). On day 7, 27 patients (87%) had a BNP level less than 100 ng/L. Age was not correlated with BNP level. With regard to the BNP increase, there was no significant difference between patients receiving norepinephrine or not during the first three days, between patients mechanically ventilated or not at admission, or between two groups of WFNS scores (1 versus 2 to 5). However, the median BNP level on day 2 was significantly higher in men than in women (162 ng/L versus 106 ng/L, *P *< 0.05). In addition, the median BNP was significantly lower in the Fisher group 1 to 2 than in group 3 to 4 on day 2 (100 ng/L versus 144 ng/L, *P *< 0.05), on day 3 (63 ng/L versus 124 ng/L, *P *< 0.05), and on day 4 (42 ng/L versus 135 ng/L, *P *< 0.05).

### Echocardiography, filling pressure and diastolic function

**Figure 4:**
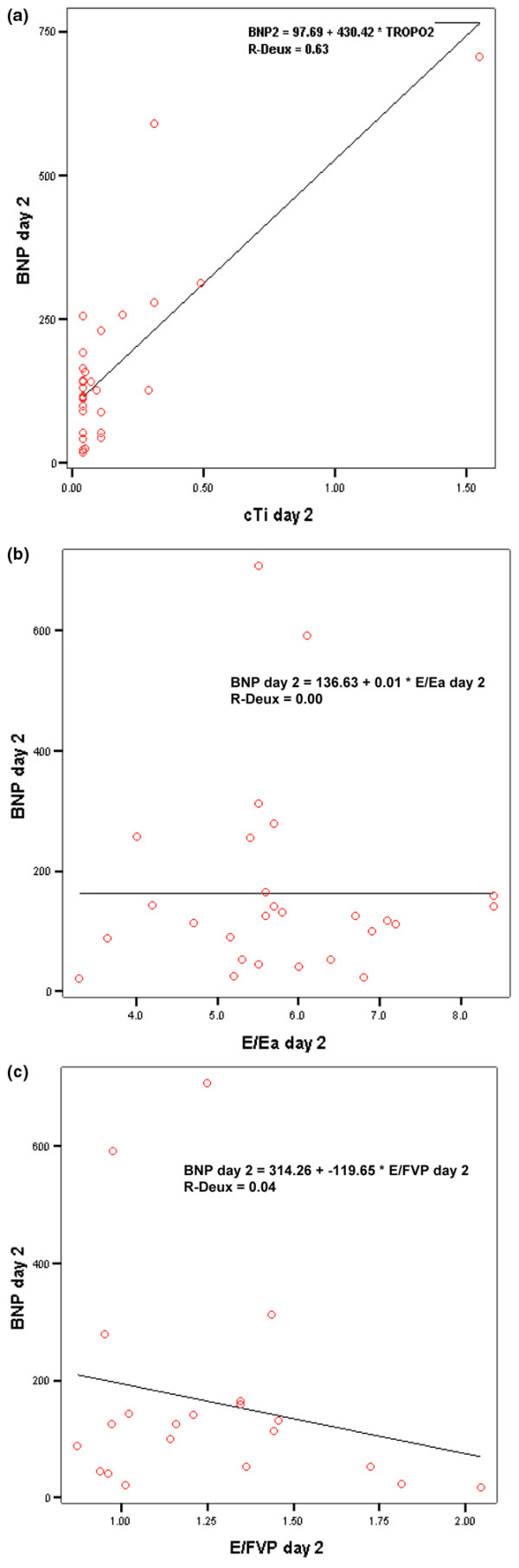
Correlations on day 2 between BNP, cTi, E/Ea, and E/FPV  **(a) **B-type natriuretic peptide (BNP) vs troponin Ic (cTi); **(b) **BNP vs early transmitral velocity (E)/tissue Doppler imaging early diastolic velocity (Ea); **(c) **E/color M-mode-derived flow propagation velocity (FPV).

Doppler echocardiographic variables are listed in Table 4. Mitral inflow were recorded in all patients and tissue Doppler imaging signals in 27 patients, but FPV recordings were considered inadequate in eight patients (26%) because of inadequate signal. All 31 patients had LVEF more than 50%. Of the 31 patients in the study, E/Ea was less than 8 in 30 patients (97%) on day 1, in 29 patients (94%) on day 2, and in all patients on day 7. Of the 23 patients recorded, E/FPV was less than 1.5 in 21 patients (87%). In all patients, DT was more than 130 ms and IRVT was more than 50 ms in the three echocardiographic exams. On days 1, 2, and 7, there were no correlations between BNP and the following echocardiographic parameters: mitral E/A, E/Ea, E/FPV, DT, PAP, LV mass, blood pressure, and IVRT (Figure 4). No significant differences in echocardiographic data were observed between the three study days. Of the seven patients (23%) with E/A less than 1 on day 1, five had the same mitral inflow profile on days 2 and 7. The other two patients had E/A more than 1 on day 7.

**Table 4:** Hemodynamic characteristics and Doppler parameters (mean ± standard deviation)

	Day 1	Day 2	Day 7
Systolic BP, mmHg	128 (± 10)	136 (± 11)	132 (± 29)
Diastolic BP, mmHg	61 (± 7)	65 (± 7)	67 (± 8)
Heart rate, beats/min	76 (± 11)	76 (± 11)	82 (± 10)
LV ejection fraction (%)	67 (± 9)	65 (± 8)	70 (± 7)
LV end-systolic volume (ml)	31 ± 5	31 ± 3	25 ± 5
LV end-diastolic volume (ml)	94 ± 16	90 ± 14	91 ± 18
Systolic PAP, mmHg	19 (± 9)	16 (± 9)	16 (± 8)
LV mass (g/m^2^)	111 (± 25)	-	-
Mitral E (cm/s)	88 (± 21)	96 (± 22)	83 (± 16)
Mitral A (cm/s)	67 (± 20)	68 (± 20)	70 (± 20)
Mitral E/A ratio	1.32 (± 0.4)	1.47 (± 0.43)	1.29 (± 0.38)
Ea, lateral annulus (cm/s)	17.1 (± 6.5)	17.2 (± 4.8)	16.3 (± 3.2)
E/Ea	5.4 (± 1.5)	5.8 (± 1.2)	5.1 (± 0.9)
FPV (cm/s)	74 (± 15)	77 (± 12)	77 (± 10)
E/FPV	1.27 (± 0.4)	1.25 (± 0.3)	1.1 (± 0.2)
Deceleration time (ms)	206 (± 50)	206 (± 37)	200 (± 39)
Isovolumic relaxation time (ms)	79 (± 21)	77 (± 17)	77 (± 19)

A = late transmitral velocity; BP = blood pressure; E = early transmitral velocity; Ea = tissue Doppler imaging early diastolic velocity; FPV = color M-mode-derived flow propagation velocity; LV = left ventricular; PAP = pulmonary artery pressure.

### Troponin Ic

The cTi level ranged from 0 to 3.67 μg/L. The proportion of patients with cTi more than 0.14 μg/L was higher on the first two days (22%, n = 7) than during the following days: 6% (n = 2) on days 3 and 4, and only 3% (n = 1) on and after day 5. The BNP level was higher in patients with cTi more than 0.14 μg/L, and the difference on day 2 was significant (106 ng/L versus 345 ng/L) (Table 5). There was a significant correlation between the cTi level on day 2 and the BNP level on day 2 (*r *= 0.63, *P *< 10^-3^; Figure 4.) and on day 3 (*r *= 0.44, *P *< 0.05). The three patients with cTi more than 0.9 μg/L presented an interesting BNP time course. Only in these cases, the peak BNP level was more than 300 ng/L. Of the four patients with BNP more than 100 ng/L on day 7, three had a cTi more than 0.9 μg/L on day 1. There were no significant differences in cTi levels between patients receiving norepinephrine or not during the first three days, between patients mechanically ventilated or not at admission, between two groups of WFNS score (1 versus 2 to 5), between men and women, or between Fisher group 1 to 2 and group 3 to 4.

**Table 5:** BNP level with or without myocardial necrosis during the first seven days (mean ± standard deviation)

	BNP day 1	BNP day 2	BNP day 3	BNP day 4	BNP day 5	BNP day 6	BNP day 7
cTi <0.14 μg/L (n = 24)	72 (± 66)	106 (± 66)	119 (± 104)	116 (± 92)	70 (± 98)	53 (± 42)	51 (± 73)
cTi = 0.14 μg/L (n = 7)	116 (± 128)	345 (± 221)*	271 (± 323)	113 (± 97)	111 (± 113)	122 (± 130)	101 (± 129)

*P < 0.001 (Mann-Whitney test)

BNP = B-type natriuretic peptide; cTi = troponin Ic.

## Discussion

To our knowledge, this is the first study focused on cardiac injury due to aneurysmal SAH in patients without pre-existing chronic hypertension and cardiac disease. The present study demonstrated that 80% of patients develop a BNP level of more than 100 ng/L during the first three days (peak on day 2) after admission for aneurysm rupture, with a return to normal levels in less than one week. Nevertheless, contrary to our hypothesis, the BNP rise was not triggered by an elevation in echo-estimated LVFP due to diastolic dysfunction. Moreover, BNP and cTi seem to be more sensitive to cardiac stress occurring in SAH as compared with Doppler variables of diastolic function.

Strong arguments favor the idea that BNP elevation is the result of intrinsic conditions of SAH but not the result of iatrogenic volume overload, especially during the first three days: standardized protocol without prophylactic hypervolemia, negative fluid balance, and near-zero sodium balance. Moreover, the rise of the BNP level during the first three days could not be influenced by the vasospasm because all cases have occurred after day 5. Our study is adds to the information from prior publications for two reasons. First, previous works have only assessed diastolic function (categorized as normal, impaired relaxation, pseudonormal, or restrictive) but not echo-estimated LVFP [10,14]. Second, patients with a history of hypertension were not excluded in these studies, in contrast to ours. They found that patients with a history of hypertension had higher mean BNP levels than patients without hypertension and a higher frequency of diastolic dysfunction, with no possibility to differentiate between BNP elevation caused by chronic cardiac abnormalities and BNP related to acute neurocardiac injury [6].

Although we did not use a pulmonary artery catheter, indices of cardiac filling pressures (E/Ea and E/FPV) were not elevated in our subjects on days 1, 2, and 7 (Table 4) and were not correlated with BNP levels. In fact, it has been clearly demonstrated that mitral E/Ea less than 8 and E/FPV less than 1.5 accurately predict normal LVFP [4,5]. Although no previous work has studied filling pressure in this type of population, other researchers have reported hemodynamic findings in line with our findings in SAH patients by using a pulmonary artery catheter placed for SAH management, mainly to prevent or treat cerebral vasospasm. The studies did not find elevated pulmonary artery wedge pressure (PAWP) during the first week after aneurysm rupture in patients without cardiac failure [18-21]. Mayer and colleagues [20] did not find elevated PAWP (12.4 ± 3.5 mmHg), although patients had been managed in a mildly volume-expanded state.

BNP is not only synthesized in response to cardiac mechanical stretch [22]. The precursor of BNP is released during myocyte stress concerning the LV or the right ventricle: heart failure (when the ventricles are dilated, hypertrophic, or subject to increased wall tension), acute coronary syndromes, pulmonary disease (e.g., acute respiratory distress syndrome, lung disease with right heart failure), pulmonary embolism, high output states (e.g., sepsis, cirrhosis, hyperthyroidism), and atrial fibrillation [23].That is why the BNP level lacks specificity in critical care patients [24].

Recently, BNP was established as a sensitive prognostic parameter in patients with acute coronary syndromes [25] and even in asymptomatic persons [26]. In addition, transient myocardial ischemia results in an immediate increase in BNP [27,28]. Furthermore, the magnitude of the increase is proportional to the severity of ischemia [28]. Tung and colleagues [10] reported a correlation between levels of BNP and troponin release during SAH, and our findings are consistent with that study. In fact, we found a strong correlation between cTi and BNP levels during the first three days after aneurysm rupture. However, we observed a BNP increase without myocardial necrosis in 65% of our patients. Several studies have reported that BNP is not only increased in necrotic myocardial tissue but also in non-necrotic myocardial tissue, such as in unstable angina, and that BNP levels reflect the severity of myocardial damage and thus might have diagnostic value [29,30]. Some authors have reported that elevated BNP or N-terminal pro-brain natriuretic peptide levels are sensitive and specific parameters for ischemia diagnosis [27,31,32]. Foote and colleagues [27] reported that the BNP level is a marker of inducible ischemia that is twice as sensitive for the detection of ischemia than is ST-segment depression on exercise electrocardiography. Bassan and colleagues [33] reported that plasma BNP is an early marker of acute myocardial infarction in patients with chest pain and non-diagnostic ECG, particularly if initial creatine-kinase MB and/or troponin Ic are non-diagnostic [33]. In these studies, the cut-off value of BNP for myocardial events was about 100 pg/mL, similar to our BNP levels [28,33].

It is tempting to extrapolate these results to myocardial injury related to SAH. However, the time course of BNP release and the mechanisms of myocardial damage are different. Reduced regional myocardial blood flow leads to myocardial ischemia with a cascade of changes, during which BNP could be an early marker to detect reduced myocardial blood flow [27,33]. It was not the case in our study where BNP rise did not occur earlier. Moreover, SAH patients have cardiac injury with normal myocardial perfusion, without angiographic evidence of coronary artery disease or vasospasm [34-36], and without myocardial hypoperfusion at the epicardial or microvascular level [37]. However, the most likely cause of cardiac dysfunction after SAH is excessive catecholamine release within the myocardium. Masuda and colleagues [37] demonstrated extremely enhanced sympathetic activity and a massive release of catecholamines from the terminals of sympathetic nerves. Massive increase in myocardial tissue occurs [36,37], but serum catecholamine levels remain relatively unchanged, without correlation with cTi [36]. Catecholamine and hemodynamic parameters (heart rate, arterial pressure) peak at five minutes and return to baseline at 30 minutes [37]. It is therefore logical to assume that these effects had disappeared at the time of admission of patients several hours after the rupture of the aneurysm. The absence of increase in E/Ea and E/FPV does not exclude cardiac injury mediated by catecholamine release. Actually, it is believed that high interstitial concentrations of norepinephrine result in myocyte calcium overload and cell death [36]. This local phenomenon could explain the delayed secretion of BNP.

It has been suggested that the pathophysiology of neurogenic cardiac injury after SAH is probably similar to apical ballooning syndrome (ABS) (Tako-Tsubo or stress cardiomyopathy) [36,38]. Although there are very few reports of BNP levels during ABS, the published results are similar to our findings in many aspects. First, a marked increase in BNP has been observed in ABS [39-41]. Second, the BNP rise is not triggered by an elevation in LVFP. In two different studies, Akashi and colleagues [39,40] reported an increase in BNP to mean values of 522.5 pg/ml and 629.6 pg/ml, respectively, whereas the LVFPs were low. Third, the BNP release kinetics observed in the case report of Nef and colleagues [41] were in complete agreement with our findings with a delayed peak in serum NT-proBNP level observed 24 hours after the onset of clinical symptoms. In most patients, BNP levels returned to normal within one week [39-41].

### Limitations section

The present study is notable in that it consists of carefully selected consecutive patients from a single center. However, the external validity of the study is strongly reduced because of selection criteria of our patients. First, we have excluded patients admitted 48 hours after the occurrence of aneurysm rupture symptoms to observe cardiac injury that occurs and develops imediately after it. It is well known that a delay in referral to neurosurgical hospital is frequent, which could potentially lead to a lag in the BNP and diastolic profile. Second, this patient selection does not allow extension of the results to all SAH patients, who are frequently hypertensive, and have higher BNP levels than patients without hypertension and a higher frequency of diastolic dysfunction [10]. Although it is possible that patients slightly or recently hypertensive have been included (unknown hypertension), our results show strong arguments to say that they did not have chronic hypertensive heart disease (diastolic dysfunction) at the time of admission considering the normal renal function on day 2, and echographic data and BNP levels normal on day 7.

## Conclusions

Using Doppler echocardiography, this study demonstrates that BNP rises gradually over two days and returns to normal within a week after SAH, without echo-estimated LVFP elevation. It provides novel evidence that levels of BNP and troponin Ic are correlated. Furthermore, the kinetics of BNP release appears to be close to those observed in ABS, which could provide an additional argument that cardiac injury is catecholamine-induced during SAH.

## Key messages

• BNP rises gradually over two days and returns to normal within a week after SAH.

• Levels of BNP and troponin Ic are correlated in SAH patients.

• Doppler echocardiography showed that echo-estimated LVFP remains low during the first week after SAH.

## Abbreviations

A: late transmitral velocity; ABS: apical ballooning syndrome; BNP: B-type natriuretic peptide; cTi: troponin Ic; DT: deceleration time of E velocity; E: early transmitral velocity; Ea: tissue Doppler imaging early diastolic velocity; ELISA: enzyme-linked immunosorbent assay; FPV: color M-mode-derived flow propagation velocity; ICU: intensive care unit; IVRT: isovolumic relaxation time; LV: left ventricular; LVEF: left ventricular ejection fraction; LVFP: left ventricular filling pressure; PAP: pulmonary artery pressure; PAWP: pulmonary artery wedge pressure; SAH: subarachnoid hemorrhage; WFNS: World Federation of Neurosurgical Societies.

## Competing interests

The authors declare that they have no competing interests.

## Authors' contributions

EM conceived, designed, and drafted the study. CJ performed all the echocardiographies. NK and AM made the collection of data and contributed to their analysis. HB performed the statistical analysis with Dr A. Loundou (see acknowledgements). PG made substantial contributions to conception and design. GH revised the manuscript critically for intellectual content. BP gave final approval of the version to be published. All authors read and approved the final manuscript.

## References

[B1] Lubien E, DeMaria A, Krishnaswamy P, Clopton P, Koon J, Kazanegra R, Gardetto N, Wanner E, Maisel AS (2002). Utility of B-natriuretic peptide in detecting diastolic dysfunction: comparison with Doppler velocity recordings. Circulation.

[B2] Tokola H, Hautala N, Marttila M, Magga J, Pikkarainen S, Kerkelä R, Vuolteenaho O, Ruskoaho H (2001). Mechanical load-induced alterations in B-type natriuretic peptide gene expression. Can J Physiol Pharmacol.

[B3] Nagueh SF, Middleton KJ, Kopelen HA, Zoghbi WA, Quinones MA (1997). Doppler tissue imaging: a noninvasive technique for evaluation of left ventricular relaxation and estimation of filling pressures. J Am Coll Cardiol.

[B4] Ommen SR, Nishimura RA, Appleton CP, Miller FA, Oh JK, Redfield MM, Tajik AJ (2000). Clinical utility of Doppler echocardiography and tissue Doppler imaging in the estimation of left ventricular filling pressures: A comparative simultaneous Doppler-catheterization study. Circulation.

[B5] Rivas-Gotz C, Manolios M, Thohan V, Nagueh SF (2003). Impact of left ventricular ejection fraction on estimation of left ventricular filling pressures using tissue Doppler and flow propagation velocity. Am J Cardiol.

[B6] Schillinger M (2005). Brain natriuretic peptide and early cardiac dysfunction after subarachnoid hemorrhage. Stroke.

[B7] Sviri GE, Shik B, Raz B, Soustiel JF (2001). Brain natriuretic peptide and cerebral vasospasm in subarachnoid hemorrhage. Acta Neurochir Suppl.

[B8] Tomida M, Muraki M, Uemura K, Yamasaki K (1998). Plasma concentrations of brain natriuretic peptide in patients with subarachnoid hemorrhage. Stroke.

[B9] Naval NS, Stevens RD, Mirski MA, Bhardwaj A (2006). Controversies in the management of aneurysmal subarachnoid hemorrhage. Crit Care Med.

[B10] Tung PP, Olmsted E, Kopelnik A, Banki NM, Drew BJ, Ko N, Lawton MT, Smith W, Foster E, Young WL, Zaroff JG (2005). Plasma B-type natriuretic peptide levels are associated with early cardiac dysfunction after subarachnoid hemorrhage. Stroke.

[B11] Zaroff JG, Rordorf GA, Newell JB, Ogilvy CS, Levinson JR (1999). Cardiac outcome in patients with subarachnoid hemorrhage and electrocardiographic abnormalities. Neurosurgery.

[B12] Tung P, Kopelnik A, Banki N, Ong K, Ko N, Lawton MT, Gress D, Drew B, Foster E, Parmley W, Zaroff J (2004). Predictors of neurocardiogenic injury after subarachnoid hemorrhage. Stroke.

[B13] Horowitz MB, Willet D, Keffer J (1998). The use of cardiac troponin-I (cTnI) to determine the incidence of myocardial ischemia and injury in patients with aneurysmal and presumed aneurysmal subarachnoid hemorrhage. Acta Neurochir (Wien).

[B14] Kopelnik A, Fisher L, Miss JC, Banki N, Tung P, Lawton MT, Ko N, Smith WS, Drew B, Foster E, Zaroff J (2005). Prevalence and implications of diastolic dysfunction after subarachnoid hemorrhage. Neurocrit Care.

[B15] Qureshi AI, Suri MF, Yahia AM, Suarez JI, Guterman LR, Hopkins LN, Tamargo RJ (2001). Risk factors for subarachnoid hemorrhage. Neurosurgery.

[B16] Beydon L (2005). Severe subarachnoid haemorrhage. Ann Fr Anesth Reanim.

[B17] Garcia MJ, Ares MA, Asher C, Rodriguez L, Vandervoort P, Thomas JD (1997). An index of early left ventricular filling that combined with pulsed Doppler peak E velocity may estimate capillary wedge pressure. J Am Coll Cardiol.

[B18] Mayer SA, Sherman D, Fink ME, Homma S, Solomon RA, Lennihan L, Beckford A, Klebanoff LM (1995). Noninvasive monitoring of cardiac output by Doppler echocardiography in patients treated with volume expansion after subarachnoid hemorrhage. Crit Care Med.

[B19] Mayer SA, Solomon RA, Fink ME, Lennihan L, Stern L, Beckford A, Thomas CE, Klebanoff LM (1998). Effect of 5% albumin solution on sodium balance and blood volume after subarachnoid hemorrhage. Neurosurgery.

[B20] Mayer SA, Lin J, Homma S, Solomon RA, Lennihan L, Sherman D, Fink ME, Beckford A, Klebanoff LM (1999). Myocardial injury and left ventricular performance after subarachnoid hemorrhage. Stroke.

[B21] Mori K, Arai H, Nakajima K, Tajima A, Maeda M (1995). Hemorheological and hemodynamic analysis of hypervolemic hemodilution therapy for cerebral vasospasm after aneurysmal subarachnoid hemorrhage. Stroke.

[B22] LaPointe MC (2005). Molecular regulation of the brain natriuretic peptide gene. Peptides.

[B23] Daniels LB, Maisel AS (2007). Natriuretic peptides. J Am Coll Cardiol.

[B24] Christenson RH (2008). What is the value of B-type natriuretic peptide testing for diagnosis, prognosis or monitoring of critically ill adult patients in intensive care?. Clin Chem Lab Med.

[B25] de Lemos JA, Morrow DA, Bentley JH, Omland T, Sabatine MS, McCabe CH, Hall C, Cannon CP, Braunwald E (2001). The prognostic value of B-type natriuretic peptide in patients with acute coronary syndromes. N Engl J Med.

[B26] Levine RJ, Maynard SE, Qian C, Lim KH, England LJ, Yu KF, Schisterman EF, Thadhani R, Sachs BP, Epstein FH, Sibai BM, Sukhatme VP, Karumanchi SA (2004). Plasma natriuretic peptide levels and the risk of cardiovascular events and death. N Engl J Med.

[B27] Foote RS, Pearlman JD, Siegel AH, Yeo KT (2004). Detection of exercise-induced ischemia by changes in B-type natriuretic peptides. J Am Coll Cardiol.

[B28] Sabatine MS, Morrow DA, de Lemos JA, Omland T, Desai MY, Tanasijevic M, Hall C, McCabe CH, Braunwald E (2004). Acute changes in circulating natriuretic peptide levels in relation to myocardial ischemia. J Am Coll Cardiol.

[B29] Talwar S, Squire IB, Downie PF, Davies JE, Ng LL (2000). Plasma N terminal pro-brain natriuretic peptide and cardiotrophin 1 are raised in unstable angina. Heart.

[B30] Kikuta K, Yasue H, Yoshimura M, Morita E, Sumida H, Kato H, Kugiyama K, Ogawa H, Okumura K, Ogawa Y, Nakao K (1996). Increased plasma levels of B-type natriuretic peptide in patients with unstable angina. Am Heart J.

[B31] Palazzuoli A, Deckers J, Calabrò A, Campagna MS, Nuti R, Pastorelli M, Pasqui AL, Bruni F, Auteri A, Puccetti L (2006). Brain natriuretic peptide and other risk markers for outcome assessment in patients with non-ST-elevation coronary syndromes and preserved systolic function. Am J Cardiol.

[B32] Hong SN, Yoon NS, Ahn Y, Lim SY, Kim YS, Yun KH, Kang DK, Lee SH, Lee YS, Kim KH, Son IS, Hong YJ, Park HW, Kim JH, Jeong MH, Cho JG, Park JC, Kang JC (2005). N-terminal pro-B-type natriuretic Peptide predicts significant coronary artery lesion in the unstable angina patients with normal electrocardiogram, echocardiogram, and cardiac enzymes. Circ J.

[B33] Bassan R, Potsch A, Maisel A, Tura B, Villacorta H, Nogueira MV, Campos A, Gamarski R, Masetto AC, Moutinho MA (2005). B-type natriuretic peptide: a novel early blood marker of acute myocardial infarction in patients with chest pain and no ST-segment elevation. Eur Heart J.

[B34] Samuels MA (1987). Neurogenic heart disease: a unifying hypothesis. Am J Cardiol.

[B35] Kono T, Morita H, Kuroiwa T, Onaka H, Takatsuka H, Fujiwara A (1994). Left ventricular wall motion abnormalities in patients with subarachnoid hemorrhage: neurogenic stunned myocardium. J Am Coll Cardiol.

[B36] Banki NM, Kopelnik A, Dae MW, Miss J, Tung P, Lawton MT, Drew BJ, Foster E, Smith W, Parmley WW, Zaroff JG (2005). Acute neurocardiogenic injury after subarachnoid hemorrhage. Circulation.

[B37] Masuda T, Sato K, Yamamoto S, Matsuyama N, Shimohama T, Matsunaga A, Obuchi S, Shiba Y, Shimizu S, Izumi T (2002). Sympathetic nervous activity and myocardial damage immediately after subarachnoid hemorrhage in a unique animal model. Stroke.

[B38] Prasad A, Lerman A, Rihal CS (2008). Apical ballooning syndrome (Tako-Tsubo or stress cardiomyopathy): A mimic of acute myocardial infarction. Am Heart J.

[B39] Akashi YJ, Nakazawa K, Sakakibara M, Miyake F, Sasaka K (2002). Reversible left ventricular dysfunction "takotsubo" cardiomyopathy related to catecholamine cardiotoxicity. J Electrocardiol.

[B40] Akashi YJ, Musha H, Nakazawa K, Miyake F (2004). Plasma brain natriuretic peptide in takotsubo cardiomyopathy. QJM.

[B41] Nef HM, Möllmann H, Weber M, Deetjen A, Brandt R, Hamm CW, Elsässer A (2007). Release pattern of cardiac biomarkers in left ventricular apical ballooning. Int J Cardiol.

